# Impact of functional mandibular advancement appliances on the temporomandibular joint - a systematic review

**DOI:** 10.4317/medoral.21180

**Published:** 2016-07-31

**Authors:** Laura Ivorra-Carbonell, José-María Montiel-Company, José-Manuel Almerich-Silla, Vanessa Paredes-Gallardo, Carlos Bellot-Arcís

**Affiliations:** 1 Dentistry graduate, Stomatology Department, Faculty of Medicine and Dentistry, University of Valencia, Spain; 2 Teaching Assistant, Stomatology Department, Faculty of Medicine and Dentistry, University of Valencia, Spain; 3 Tenured Lecturer, Stomatology Department, Faculty of Medicine and Dentistry, University of Valencia, Spain; 4 Associate Lecturer, Stomatology Department, Faculty of Medicine and Dentistry, University of Valencia, Spain

## Abstract

**Background:**

Although many orthodontists have no doubts about the effectiveness of functional appliances for mandibular advancement, the impact on the temporomandibular joint (TMJ) is still in dispute. The objective of this systematic review is to examine the main effects on the TMJ of using functional appliances, both in healthy patients and in patients with a pre-existing disorder.

**Material and Methods:**

A systematic review of the literature was conducted in accordance with the PRISMA guidelines. Only systematic reviews, meta-analyses, randomized clinical trials (RCTs), case-control studies and cohort studies were included. A detailed language-independent electronic search was conducted in the Pubmed, Scopus, Cochrane Library and Embase databases. All studies published between 2000 and 2015 were included.

**Results:**

A total of 401 articles were identified. Of these, 159 were duplicates and were excluded. On reading the title and abstract, 213 articles were excluded because they did not answer the research question, leaving a total of 29 articles. These articles were read and assessed. Following critical reading of the full text, eight articles were excluded: seven because they were considered of low quality and one because it published redundant data. As a result, 21 articles were included.

**Conclusions:**

After treatment with functional appliances, the condyle was found to be in a more advanced position, with remodelling of the condyle and adaptation of the morphology of the glenoid fossa. No significant adverse effects on the TMJ were observed in healthy patients and the appliances could improve joints that initially presented forward dislocation of the disk.

**Key words:**Temporomandibular joint, TMJ, orthodontic appliances, functional, mandibular advancement, herbst appliance, bionator.

## Introduction

The use of a mandibular advancement appliance to correct skeletal malocclusions associated with mandibular retrognathism is indicated during the first stage of orthodontic treatment (1). The objective is to stimulate mandibular growth and correct the sagittal misalignment by bringing the condyles forward and downward within the glenoid fossa (2,3), as well as remodelling the condyle and glenoid fossa, causing anterior rotation of the mandible and consequently projecting it forwards (4). During this first stage, mandibular retrognathism can be treated with either fixed or removable functional appliances (3,5). The treatment period lasts approximately 6 to 9 months (4).

When the functional appliance is inserted, the condyles are moved to a higher position in the articular eminence, which is capable of adaptation, so it could be hypothesized that some morphological changes may take place (6).

Although many orthodontists have no doubts about the effectiveness of functional appliances, their impact on the TMJ is still considered a subject of debate. It is therefore of clinical and scientific interest to investigate the most relevant effects of different functional appliances on the TMJ, particularly with current diagnostic methods. Many authors assert that treatment with these devices does not increase the prevalence of temporomandibular disorders (1,7-9). Another important aspect is the positive or negative effect that functional appliances may have on the TMJ of patients with a preexisting disorder (1).

The main objective of this systematic review is to examine the main effects on the TMJ of different functional appliances for mandibular advancement, both in healthy patients and in patients with a pre-existing disorder.

## Material and Methods

A systematic review of the bibliography was carried out in accordance with the PRISMA (Preferred Reporting Items for Systematic Reviews and Meta-Analyses) recommendations (10) and CONSORT criteria (11).

- Eligibility criteria

The selection criteria for inclusion in the review were: articles, articles in press and reviews. Only the following types of study were accepted: systematic reviews, meta-analyses, randomized clinical trials (RCTs), case-control studies and cohort studies. Both retrospective and prospective studies were included. The articles that met these criteria and studied changes in the temporomandibular joint following the use of functional appliances for mandibular advancement were included in the review.

- Search strategy and screening

To identify the relevant studies, irrespective of language, a thorough electronic search was conducted in the Pubmed, Scopus, Cochrane Library and Embase databases. All studies published between 2000 and 2015 were included. The search was updated on 4 July 2015.

The search strategy was implemented through a combination of the following 6 MeSH terms: “Temporomandibular joint”, “TMJ”, “Functional orthodontic appliances”, “Mandibular advancement”, “Herbst appliance”, “Bionator” and “Activator appliances”, and others: “Fränkel Function Regulator”, “Orthodontic Appliances”, “Activator”, “Function Activator”, “Bionator Appliance”, “Functional Orthodontic” and “Twin Block”. The TMJ related terms were combined using the OR Boolean operator, as well as those related to the mandibular advancement devices. Both groups were combined using the AND Boolean operator. Hand-searching in the reference lists of the articles assessed for the review was also carried out.

Two independent reviewers assessed the titles and abstracts of all the articles selected. In the event of their disagreeing, a third reviewer was consulted. If the abstract did not provide sufficient information for a decision, the reviewers read the full article before taking the final decision. The reviewers then read the full text of all the resulting articles.

- Data extraction

For each article assessed, the following variables were recorded: author, year published, type of study (retrospective, prospective, controlled, not controlled), sample size, dropouts, demographic variables (gender, age), type of appliance used, type of advancement (sequential or otherwise), presence or otherwise of preexisting TMJ disorders, length of treatment, type of radiographic study used to study the changes, follow-up time for each study and quality of the articles accepted ( Table 1). The articles were classified as being of high, medium or low quality according to the CONSORT criteria (11) adapted by Mattos *et al.* (12) and used by Fernández-Ferrer *et al.* and Serra-Torres *et al.* (13,14). The quality of the systematic reviews was assessed in accordance with the PRISMA guidelines (10).

**Table 1:**
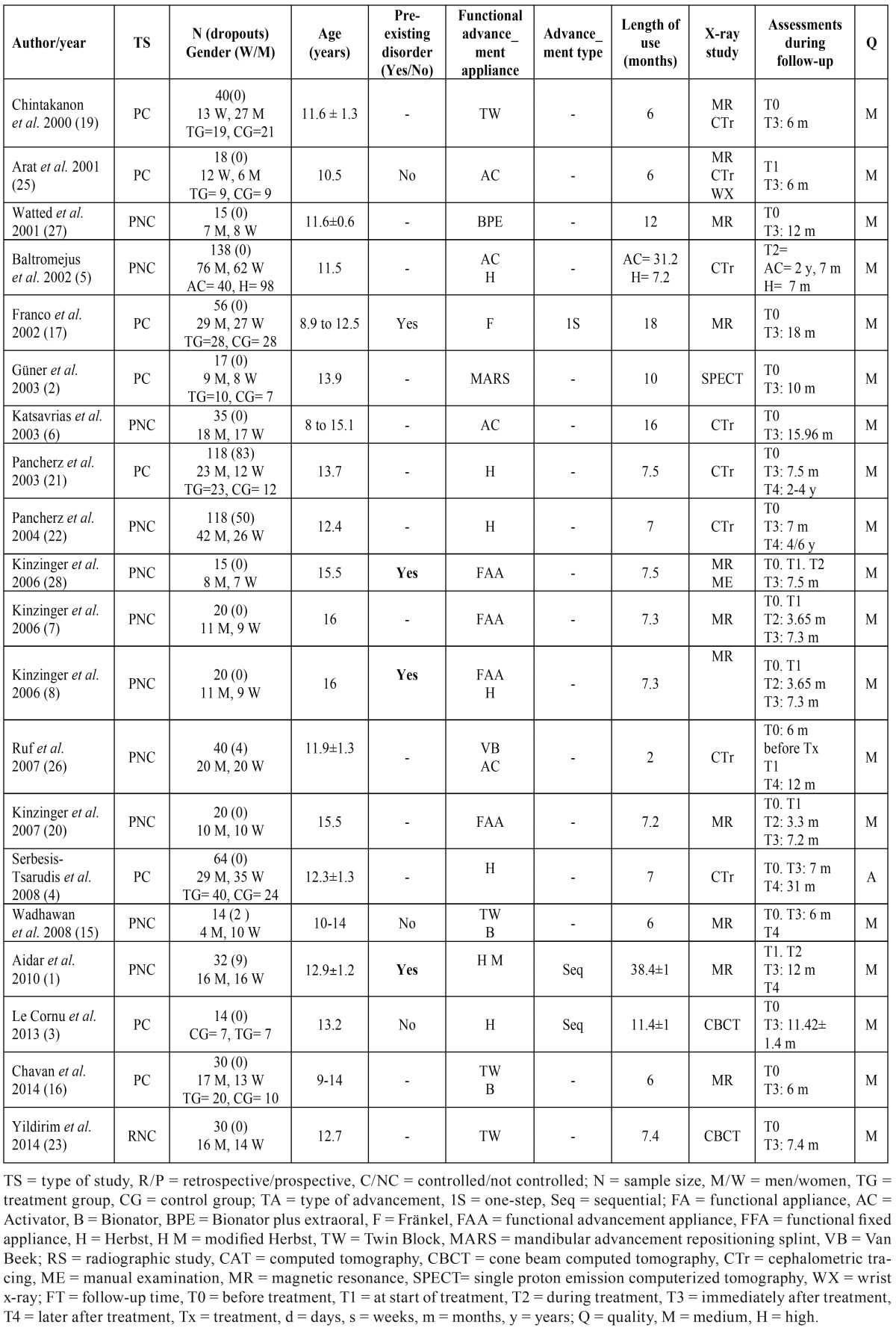
Articles included, with information from each.

## Results

**Figure 1:**
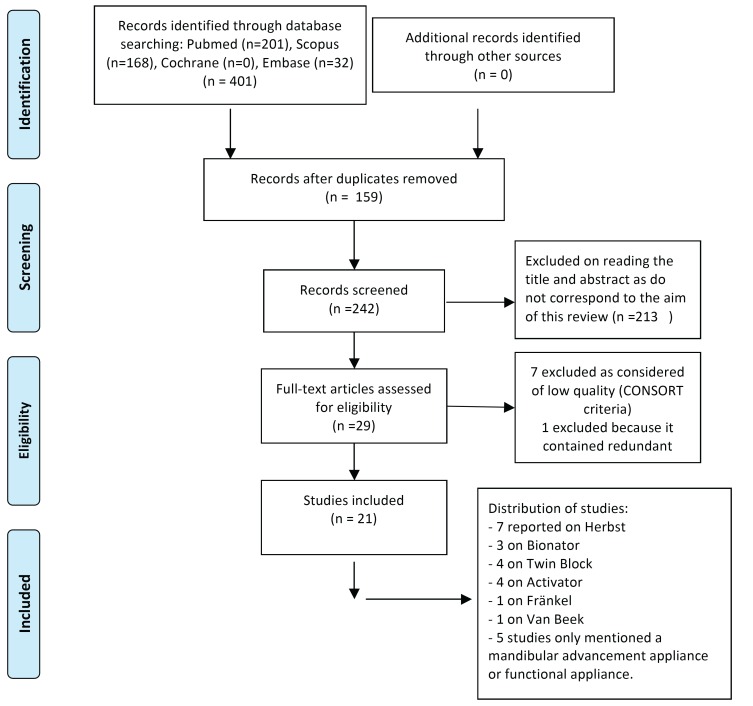
Flow diagram.

A thorough search identified 201 articles in Medline, 168 in Scopus, none in the Cochrane Library and 32 in Embase, making a total of 401 articles. 159 duplicates were removed, leaving 242. After reading the title and abstract, 213 were removed because they did not answer the research question, leaving a total of 29 articles. These were read and carefully assessed. Detailed critical reading of the full text resulted in the exclusion of 8 articles, 7 because of their low quality (Fig. 1) and 1 because it published redundant data. Consequently, 21 articles that met all the inclusion criteria and were of medium to high quality were included in the review. It should be mentioned that the manual search did not identify any relevant articles.

Regarding the type of study reported in the 21 articles, 1 was a systematic review, 1 was a retrospective study and 19 were prospective studies. Of the prospective studies, 8 were controlled and 11 were not controlled ( Table 1).

Concerning the state of the joint immediately prior to treatment, leaving aside the systematic review, only 8 of the studies explicitly mentioned its condition. Of these, 4 studied healthy joints and 4 studied joints with certain disorders ( Table 2). The remaining 12 studies made no mention of joint status.

**Table 2:**
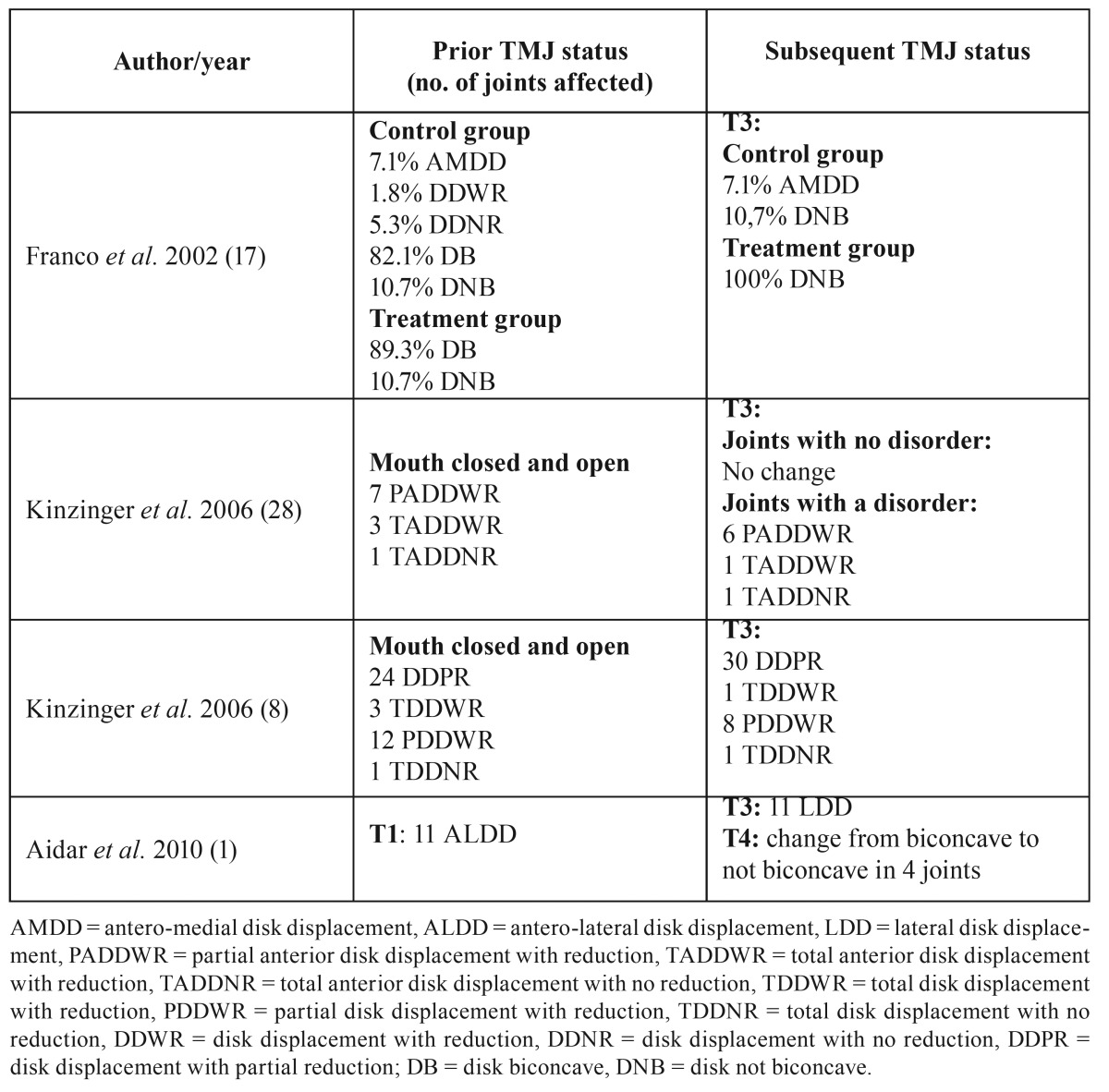
Articles included, with information on pre-existing conditions.

With regard to the type of appliance used, 7 studies used the Herbst appliance. Of these, 5 did not use control groups, 1 compared it with a functional advancement device and 1 with the Activator. The Bionator was studied in 3 articles, comparing it with the Twin Block in 2 cases. Two studies concerned the Twin Block alone, a further two the Activator alone and one the Fränkel, while another compared the Activator with the Van Beek. Three other articles did not name the type of appliance, mentioning only a functional or mandibular advancement appliance.

Only three studies specified the type of mandibular advancement (sequential or one-step).

In relation to the type of diagnostic study, 8 used magnetic resonance, 2 CBCT, 1 SPECT (single-proton emission computed tomography), 6 cephalometric tracing, 1 magnetic resonance together with manual examination, 1 magnetic resonance as well as cephalometric tracing and wrist radiographs, and 1 used magnetic resonance and teleradiography.

Regarding the quality of the articles, 19 were of moderate quality and one of high quality. The systematic review was considered of high quality. The main conclusions drawn by the authors of the studies included in the present review are shown in  Table 3.

**Table 3:**
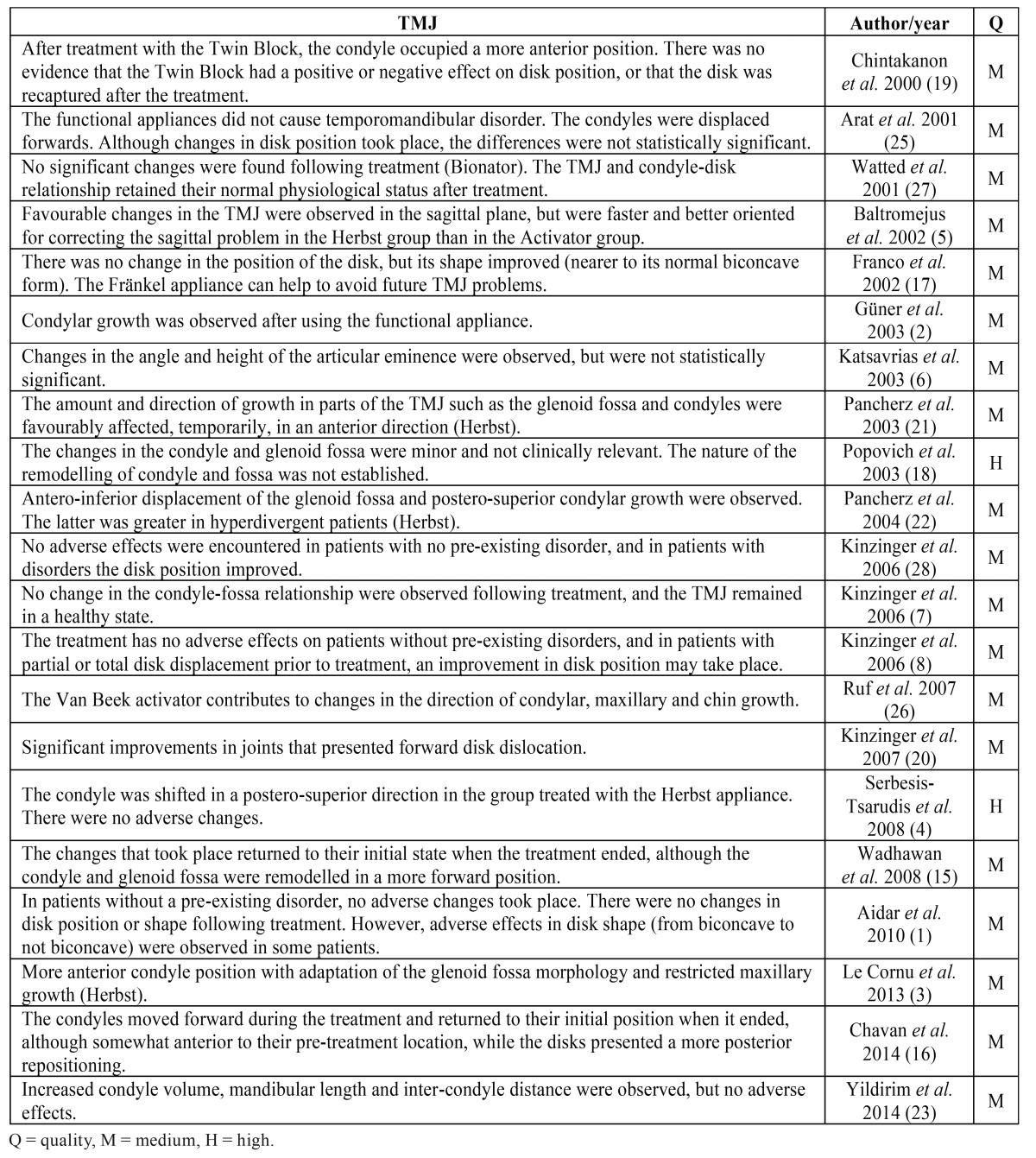
Studies by observations.

## Discussion

- Changes in disk shape and position

According to Wadhawan *et al.* (15), the anterior repositioning of the condyle in the glenoid fossa caused by functional appliances results in stretching of the retrodiscal tissue that could lead to changes in the shape of the disk or its posterior displacement, which agrees with the study by Chavan *et al.* (16). However, Aidar *et al.* (1) found no significant changes in disk position after treatment with the Herbst appliance. Franco *et al.* (17) observed no changes in comparison with the control group whether in healthy patients or in patients with antero-medial disk displacement, coinciding with the systematic review by Popowich *et al.* (18) and the study by Chintakanon *et al.* (19). Aidar *et al.* (9), in a previous study, made the same observation following 12 months’ use of the Herbst appliance in adolescents whose disks were within the normal limits prior to treatment. In patients with prior disk displacement, these authors observed that it was not recaptured. However, Kinzinger *et al.* (20), using magnetic resonance, observed significant improvements in joints with an initial forward disk dislocation, agreeing with Kinzinger *et al.* (8), who also observed a lack of adverse effects in patients with a good initial condyle-disk relationship, as did Katsavrias *et al.* (6). Pancherz *et al.* (21) performed the same observation and indicated that the improvement in disk position depended on the extent of the initial displacement.

Concerning disk shape, Aidar *et al.* (9) reported a lack of adverse effects on articular disk morphology. Subsequently, the same authors (1) reached the same conclusion despite finding adverse effects in some patients at the end of the treatment, such as a change in disk shape from biconcave to not biconcave. Franco *et al.* (17), using magnetic resonance, observed that at the end of the treatment the number of biconcave discs had increased significantly in the patients treated with Fränkel appliances. As a result, they indicated that the use of this appliance at the beginning of the growth stage could help avoid future intra-TMJ problems.

- Changes in condyle size and position

In a case-control study of treatment with the Herbst appliance, Le Cornu *et al.* (3) observed forward adaptive displacement of the condyles followed by remodelling of the glenoid fossa, compared with a control group that was only treated with fixed devices and class II elastic bands. These findings agree with those of Chintakanon *et al.* (19) and Chavan *et al.* (16). The latter conducted a controlled study using magnetic resonance and observed a more forward condyle position, although they recommended long-term follow-up to investigate the stability of their findings. In the same way, Pancherz *et al.* (22) observed that changes in condyle growth were greater in men than in women and highlighted that treatment with the Herbst appliance temporarily stimulated condyle growth. A year later, in a new study of 118 patients classified according to their growth pattern, Pancherz *et al.* (22) concluded that condyle growth took place and was more posterior in hyperdivergent than in hypodivergent subjects.

In contrast to the above observations, Kinzinger *et al.* (20) found no significant differences in the glenoid fossa-condyle relationship before and after treatment. Wadhawan *et al.* (15), using magnetic resonance, observed that the condyle and disk were displaced during treatment but returned to their initial position when the treatment ended. The systematic review by Popowich *et al.* (18) also concluded that the changes in condyle position were not clinically significant.

- Changes in the glenoid fossa

Pancherz *et al.* (21) observed antero-inferior displacement of the glenoid fossa as a reaction to the Herbst treatment. These authors asserted that the Herbst had an effect on the physiological growth of the fossa, temporarily displacing it forwards. However, the review by Popowich *et al.* (18) concluded that remodelling of the glenoid fossa had not been established, which agrees with the findings of Chintakanon *et al.* (19). Focusing on patients with different growth patterns, Pancherz *et al.* (22) concluded that there were no significant changes in the glenoid fossa between hyper- and hypodivergent subjects.

- Changes in the articular eminence and other changes

Reports on adaptations of the articular eminence caused by functional appliances are scarce. Katsavrias *et al.* (6) highlighted that the height of the articular eminence was not affected and that its angle suffered minor changes, but that these were insignificant.

With regard to other changes that may take place, according to the study by Le Cornu *et al.* (3) the Herbst appliance wearers exhibited restricted maxillary growth compared to the control group. Also, no great differences were found in the mandibular body and the growth of the ramus, in condyle flexibility or in changes in gonial angle. In contrast, Yildirim *et al.* (23) observed that treatment with the Twin Block increased the volume of the condyle, the length of the mandible and the inter-condyle distance due to stimulation of condyle growth upwards and backwards.

- Limitations of this review

The scientific evidence collected on changes in the TMJ following the use of functional appliances was not abundant despite the thorough systematic search for articles that met the strict inclusion criteria and presented medium to high quality according to the CONSORT criteria.

TMJ status is a particularly important subject, yet this review has shown a lack of randomized controlled trials with long-term follow-up using diagnostic tests such as magnetic resonance and computed tomography to establish the effects of the functional appliance in a reliable way.

The articles included in the review show a lack of methodological homogeneity. For instance, Aidar *et al.* (9), Kinzinger *et al.* (20) and Wadhawan *et al.* (15) used magnetic resonance as a diagnostic method, while others such as Le Cornu *et al.* (3) and Arici *et al.* (24) used CBCT. Yet others, like Baltromejus *et al.* (5) and Güner *et al.* (2), used cephalometric tracing. Many others, such as Arat *et al.* (25), preferred to use several methods to increase the accuracy of the results.

They also employed different appliances: while the great majority used the Herbst, others studied removable appliances such as Twin Block, Bionator (15,16), Fränkel (17) or Activator (25). Only one of the studies (5) compared fixed and removable appliances, namely Activator and Herbst, concluding that the Herbst appliance corrected the sagittal problems in less time than the Activator.

Additionally, the sample size was small in most of these studies: Le Cornu *et al.* (3) studied 14 patients, Ruf *et al.* (26) 40 patients and Katsavrias *et al.* (6) 35 patients. Watted *et al.* (27) and Kinzinger *et al.* (28) studied 15 patients. The study with the largest sample size was Baltromejus *et al.* (5), with 138 cases.

## Conclusions

• Following this review, it may be concluded that observations after treatment with functional appliances for mandibular advancement have found the condyle in a more advanced position, condyle remodelling and adaptation of the morphology of the glenoid fossa.

• No significant adverse events concerning the temporomandibular joint have been found in healthy patients, and this treatment could improve joints that initially presented forward disk dislocation.
